# HIV-1 Tat and cocaine mediated synaptopathy in cortical and midbrain neurons is prevented by the isoflavone Equol

**DOI:** 10.3389/fmicb.2015.00894

**Published:** 2015-09-08

**Authors:** Sarah J. Bertrand, Calvin Hu, Marina V. Aksenova, Charles F. Mactutus, Rosemarie M. Booze

**Affiliations:** Laboratory Program in Behavioral Neuroscience, Department of Psychology, University of South CarolinaColumbia, SC, USA

**Keywords:** soy isoflavone, F-actin, S-Equol, estrogen receptor, HAND

## Abstract

Illicit drugs, such as cocaine, are known to increase the likelihood and severity of HIV-1 associated neurocognitive disorders (HAND). In the current studies synaptic integrity was assessed following exposure to low concentrations of the HIV-1 viral protein Tat 1-86B, with or without cocaine, by quantifying filamentous actin (F-actin) rich structures (i.e., puncta and dendritic spines) on neuronal dendrites *in vitro*. In addition, the synapse-protective effects of either R-Equol (RE) or S-Equol (SE; derivatives of the soy isoflavone, daidzein) were determined. Individually, neither low concentrations of HIV-1 Tat (10 nM) nor low concentrations of cocaine (1.6 μM) had any significant effect on F-actin puncta number; however, the same low concentrations of HIV-1 Tat + cocaine in combination significantly reduced dendritic synapses. This synaptic reduction was prevented by pre-treatment with either RE or SE, in an estrogen receptor beta dependent manner. In sum, targeted therapeutic intervention with SE may prevent HIV-1 + drug abuse synaptopathy, and thereby potentially influence the development of HAND.

## Introduction

Cocaine abuse significantly increases the risk of HIV-1 transmission. HIV-1+ individuals that use cocaine progress from HIV-1 infection to AIDS more quickly (Baum et al., 2009), are more likely to develop neurocognitive disorders (Levine et al., 2006; Nath, 2010), and display an accelerated progression of HIV-1 associated neurocognitive disorders (HAND; Nath, 2010). Neurocognitive dysfunction and drug abuse are two of the most common factors for medication non-compliance in treatment of HIV-1 (Panos et al., 2014), and therefore HIV-1 positive drug abusers with neurocognitive disorders have a higher risk for disease progression, increased mortality rates, and a lower quality of life (Wisniewski et al., 2005; Cook et al., 2007; Baum et al., 2009; Doshi et al., 2012; Qian et al., 2014).

Effective combined anti-retroviral therapy (cART) suppresses viral replication. However, infected astrocytes and microglia may continue to release HIV-1 proteins (i.e., Tat), into the extracellular space (Nath, 2010; Desplats et al., 2013). The released HIV-1 Tat proteins may then interact with neurons to produce dysfunction (Moore et al., 2006; Chang et al., 2008; Gongvatana et al., 2011; Desplats et al., 2013; Ortega et al., 2015). Exposure to the HIV-1 Tat protein produces loss of F-actin prior to cell death (Bertrand et al., 2013, 2014). HIV-1 Tat protein increases actin depolymerization (Wu et al., 2004) and HIV-1 Tat protein alters NMDA receptor calcium currents via interactions with the actin cytoskeleton (Krogh et al., 2015). The HIV-1 Tat induced loss of F-actin may be a reversible process, mediated by stimulation of estrogen receptors (Bertrand et al., 2014). Thus, F-actin dysregulation may play a key role in HIV-1 induced synaptodendritic damage and synaptopathy.

Pre- and post-synaptic neuronal structures are rich in actin, a dynamic cytoskeletal protein (Zhang and Benson, 2001; Johnson and Ouimet, 2006; Sekino et al., 2007; Hotulainen et al., 2009). Subsequent to cellular signaling, globular actin (G-actin) is polymerized into filamentous actin (F-actin), resulting in a stable synaptic structure (Johnson and Ouimet, 2006; Sekino et al., 2007; Hotulainen et al., 2009). Stabilized synaptic structures are rich in F-actin and appear as punctate dendritic structures (Bertrand et al., 2014); these F-actin neuronal structures may be either protruding mature dendritic spines, early stages of developing dendritic spines (patches; Matus et al., 1982; Johnson and Ouimet, 2006; Sekino et al., 2007; Hotulainen et al., 2009), non-spiny or inhibitory synapses (Heller et al., 2012), or pre-synaptic structures (Halpain, 2003; Sankaranarayanan et al., 2003). Since F-actin puncta encompass a variety of synaptic structures (Halpain, 2003; Sankaranarayanan et al., 2003) decreased F-actin puncta indicates a synaptopathy in which synaptic response and connectivity is globally diminished.

HIV-1 transgenic rats have abnormally short and stubby dendritic spines (Roscoe et al., 2014). Similarly, tat protein transgenic mice show decreased spine density (Fitting et al., 2015). HIV-1 infected cells have significantly decreased spine density, spine length, spine area, and spine number in comparison to uninfected control cells (Kurapati et al., 2013) and increased HIV-1 cell death in combination with cocaine (Kurapati et al., 2014). Conversely, chronic cocaine exposure in rats increased the expression of F-actin (Kalivas, 2009) and increases dendritic spine density (Martin et al., 2011). Actively inhibiting the polymerization of G-actin into F-actin prevents spine morphological changes found in the nucleus accumbens of rats acutely treated with cocaine (Toda et al., 2010). Although it is clear that both HIV-1 Tat protein and cocaine alter dendritic spines, the combined effects of HIV-1 Tat and cocaine on F-actin containing synapses have not been investigated.

Phytoestrogens are naturally occurring compounds that are structurally similar to 17β-estradiol (Glazier and Bowman, 2001; Lephart et al., 2005). The soy-derived phytoestrogen daidzein prevents HIV-1 Tat induced neuronal apoptosis (Adams et al., 2012) and synaptodendritic injury caused by HIV-1 Tat (Bertrand et al., 2014). Daidzein is initially metabolized in the gut into dihydrodaidzein, which is further metabolized into either *O*-desmethylangolensin or *cis/trans*-isoflavan-4-ol (Lampe, 2009; Jackson et al., 2011). Finally, *cis/trans*-isoflavn-4-ol is processed into S-Equol (SE). Due to the chiral center at carbon 3, equol can exist in either the S or R conformation; however, only SE is produced by humans and animals (Setchell et al., 2005). Equol affects neuronal mitochondrial function (Yao et al., 2013) and hypothalamic neuronal expression (Patisaul et al., 2009) *in vivo*, suggesting that equol can cross the blood–brain barrier at physiological concentrations.

HIV-1+ cocaine abusers are more likely to display neurocognitive deficits relative to non-drug abusers (Levine et al., 2006; Nath, 2010); however, the mechanism producing these neurocognitive deficits is unclear. HIV-1 viral infection in the presence of cocaine has been shown to cause cell death *in vitro* (Kurapati et al., 2013, 2014). HIV-1 Tat protein + cocaine has been reported to produce enhanced levels of apoptosis (Kendall et al., 2005). In contrast, the effects of HIV-1 Tat proteins + cocaine on synapses are unknown. The present study uses the quantification of F-actin puncta, a marker of dendritic synapses, to evaluate the effects of HIV-1 Tat and cocaine. Additionally, we evaluated the estrogen receptor-mediated mechanisms of both SE and R-Equol neuroprotection. Our results suggest that HIV-1 Tat and cocaine together produce enhanced synaptodendritic injury, resulting in a synaptopathy that may contribute to the increased incidence of neurocognitive disorders in HIV-1+ cocaine users. S- and R-Equol (RE) treatment may prevent this synaptopathy via specific estrogen receptors.

## Materials and Methods

### Ethics Statement

Experiments were in accordance with NIH Guidelines. The Institutional Animal Care and Use Committee at the University of South Carolina (assurance number: A3049-01) reviewed and approved all animal usage.

### Primary Neuronal Cell Culture

Cortical and midbrain regions were dissected from gestational day 18 Sprague-Dawley rat fetuses (Harlan Laboratories, Indianapolis, IN, USA) as previously described (Aksenova et al., 2006, 2009; Bertrand et al., 2011; for protocol see Li et al., 2015). Following dissection, brain tissue was incubated in a solution of 2 mg/ml trypsin in Hank’s balanced salt solution (HBSS) buffered with 10 mM HEPES (GIBCO Life Technologies, Grand Island, NY, USA) for 10 min. Tissue was rinsed with fresh HBSS three times and then exposed to soybean trypsin inhibitor (1 mg/ml in HBSS) for 2 min. Tissue was washed three times with HBSS following trypsin inhibitor treatment. For cytomorphological studies, cells were distributed to 12 well glass-bottom dishes and 35 mm dishes (MatTek Corporation, Ashland, MA, USA) coated with poly-L-lysine following dissociation by trituration. In order to observe distinct second order branching patterns, a low plating density was used (120–140 cells/mm^2^). Initial plating media contained Dulbecco’s modified Eagle’s medium/Ham’s nutrient mixture F-12 (DMEM/F12; GIBCO) supplemented with 100 ml/L fetal bovine serum (Sigma Chemicals, St. Louis, MO, USA). DMEM/F12 and fetal serum were removed and replaced with an equal amount of serum-free Neurobasal medium after 24 h. Neurobasal medium had no phenol red and was supplemented with 2% v/v B-27, 2 mM GlutaMAX supplement and 0.5% w/v D-(1) glucose (all ingredients from GIBCO). Cultures were maintained at 37°C in a 5% CO_2_/95% room air-humidified incubator at all times. Fresh Neurobasal medium was supplemented at weekly intervals. Midbrain cultures used for experiments were 21–30 days *in vitro* (DIV), cortical cultures were used for experiments at 14–21 DIV, cell cultures from both regions were >85–90% neuronal as determined by MAP-2/GFAP/NucBlue fluorescent staining.

### Experimental Drug Treatments

Recombinant Tat 1-86B (LAI/Bru strain of HIV-1 clade B, GenBank accession no. K02013; Diatheva, Fano, Italy) was added to the serum free growth media (10 or 50 nM final concentration). In experiments where cocaine was included, freshly prepared cocaine solution (1.6 μM final concentration) was added to the serum free grown media concurrently with HIV-1 Tat 1–86B treatment. The 1.6 μM concentration of cocaine in the cultures reflects levels found in the arterial blood supply to the brain following IV cocaine administration in humans (Evans et al., 1996) and rats (Mactutus et al., 1994; Booze et al., 1997), thus representing a physiologically relevant concentration of cocaine. This cocaine concentration has been used in our previous studies (Kendall et al., 2005; Aksenov et al., 2006, 2008), and is well below the neurotoxic levels of cocaine (100 μM; Bennett et al., 1993). The low concentration of S- and RE (33 nM), as well as the mid-range concentration of S- and RE (50 nM) used in these studies, are similar to plasma concentrations observed in humans following supplementation (8 and 12 ng/ml, respectively; Jackson et al., 2011). Control cultures were treated with an equivalent volume of vehicle. Cultures were incubated with either 50 nM HIV-1 Tat, 10 nM HIV-1 Tat, 1.6 μM cocaine, or HIV-1 Tat(10 nM) + cocaine(1.6 μM) for 24 h prior to fixation.

Cortical and midbrain cell cultures were treated with either SE (final concentration 33 or 50 nM; ≥98.5% purity; Cayman Chemical, Ann Arbor, MI, USA), RE (final concentration 33 or 50 nM; ≥98.5% purity; Cayman Chemical Ann Arbor, MI, USA), or tamoxifen (TMX; selective estrogen receptor antagonist; final concentration 100 nM; TMX citrate; Tocris Bioscience, Ellisville, MD). For the neuroprotection studies, cells were treated with either SE or RE (33 or 50 nM) for 24 h prior to treatment with either 50 nM HIV-1 Tat or 10 nM HIV-1 Tat + 1.6 μM cocaine. In order to determine which estrogenic receptors were necessary for SE and RE neuroprotection, cells were treated with TMX for 1 h prior to treatment with either SE (50 nM), RE (50 nM), 4-[2-Phenyl-5,7-*bis*(trifluoromethyl)pyrazolo[1,5-*a*]pyrmidin-3-yl)phenol (PHTPP) estrogen receptor beta (ERβ) antagonist, final concentration 100 nM (Tocris Bioscience, Ellisville, MD, USA); 1,3-*Bis*(4-hydroxyphenyl)-4-methyl-5-[4-(2-piperidinylethoxy)phenol]-1*H*-pyrazole dihydrochloride (MPP) estrogen receptor alpha antagonist; final concentration 100 nM (Tocris Bioscience, Ellisville, MD, USA), or (3a*S^∗^,4R^∗^*,9b*R^∗^*)-4-(6-Bromo-1,3-benzodioxol-5-yl)-3a,4,5,9b-3*H*-cyclopenta[*c*]quinoline (G15) membrane estrogen receptor antagonist, final concentration 100 nM (Tocris Bioscience, Ellisville, MD, USA). TMX, PHTPP, G15, SE, and RE were initially dissolved in DMSO and then diluted in PBS prior to treatment. MPP was initially dissolved in distilled water, and diluted immediately prior to treatment. For neuroprotection studies, cells were treated with either SE or RE (33 or 50 nM) for 24 h prior to treatment with 50 nM Tat or 10 nM Tat and 1.6 μM cocaine. In order to determine which of the specific estrogen receptor subtypes might underlie SE and RE neuroprotection, cells were treated with TMX, PHTPP, MPP, or G15 for 1 h prior to treatment with SE or RE (50 nM).

### Fluorescent Staining and Immunocytochemistry

A previously described method (Bertrand et al., 2013; for detailed protocol see Li et al., 2015) for F-actin staining was used to visualize F-actin puncta localized on neuronal dendrites. Briefly, primary neuronal cultures were fixed using 4% paraformaldehyde and permeabilized with 0.1% Triton X-100. Cells were incubated with the F-actin specific probe, phalloidin (1:40; AlexaFluor 488), for 20 min (Invitrogen Life Technologies, Grand Island, NY, USA).

Following staining for F-actin, cultures were treated with 10% normal horse serum in PBS for 2 h. Neurons were co-labeled with chicken polyclonal anti-MAP2 antibodies (1:1000; Abcam, Cambridge, MA, USA). Goat anti-chicken IgG conjugated with Alexa Red 594 (1:500; Invitrogen Life Technologies, Grand Island, NY, USA) was used as a secondary antibody to identify MAP2-positive dendrites. NucBlue (Molecular Probes Life Technologies, Grand Island, NY, USA), a Hoescht dye, was added directly to the cultures to identify cell nuclei (Bertrand et al., 2014).

A Nikon Eclipse TE2000-E inverted fluorescent microscope (20X objective, 1600 × 1200 px image size, 0.17 μm/px image resolution at 1X zoom) and a high resolution CCD camera was used to obtain images of neurons co-labeled with F-actin/MAP-2/NucBlue. The NIS-Elements software package (Nikon Instruments, Melville, NY, USA) was used to analyze 3–5 separate neurons per culture well. All imaged neurons had clearly identifiable complete dendritic arbors and normal nuclear morphology. F-actin puncta were identified in several (3–4) second order dendritic segments (15–75 μm) for each neuron. For inclusion, dendrites were required to have continuous MAP-2 positive immunofluorescence co-localized with Phalloidin staining. Phalloidin-labeled structures included fine filopodia, spine protrusions, and patches. Growth cones (F-actin rich structures at the distal end of dendrites) were excluded (Bertrand et al., 2013).

F-actin puncta were quantified as previously described (Bertrand et al., 2013, 2014). Briefly, background fluorescence from the 488 (green phalloidin labeled F-actin) channel was subtracted, identified as low fluorescence of the non-synaptic dendritic shaft (20–50 au). Bright F-actin rich structures on the dendrite (fine filopodia, spine protrusions, and patches) were manually counted. The number of F-actin puncta per 10 μm of dendrite was reported by two trained independent observers. These investigators, blinded to experimental treatment, quantified puncta from identically processed images, with a very high correlation (*r*^2^ = 0.90), indicating that the bright green F-actin puncta on dendrites were readily identified.

### Statistical Analysis

Statistical comparisons were made using ANOVA techniques, and Tukey’s multiple comparison tests were used to determine specific treatment effects. Pearson’s product-moment correlation coefficient was calculated to verify inter-rater reliability. Comparisons and correlations were calculated using SPSS version 22 (IBM Corporation, Armonk, NY, USA). Data represents mean values ± SEM. Significant differences were set at *p* ≤ 0.05.

## Results

### S-Equol and R-Equol Prevent HIV-1 Tat Induced Synapse Loss

**FIGURE 1:**
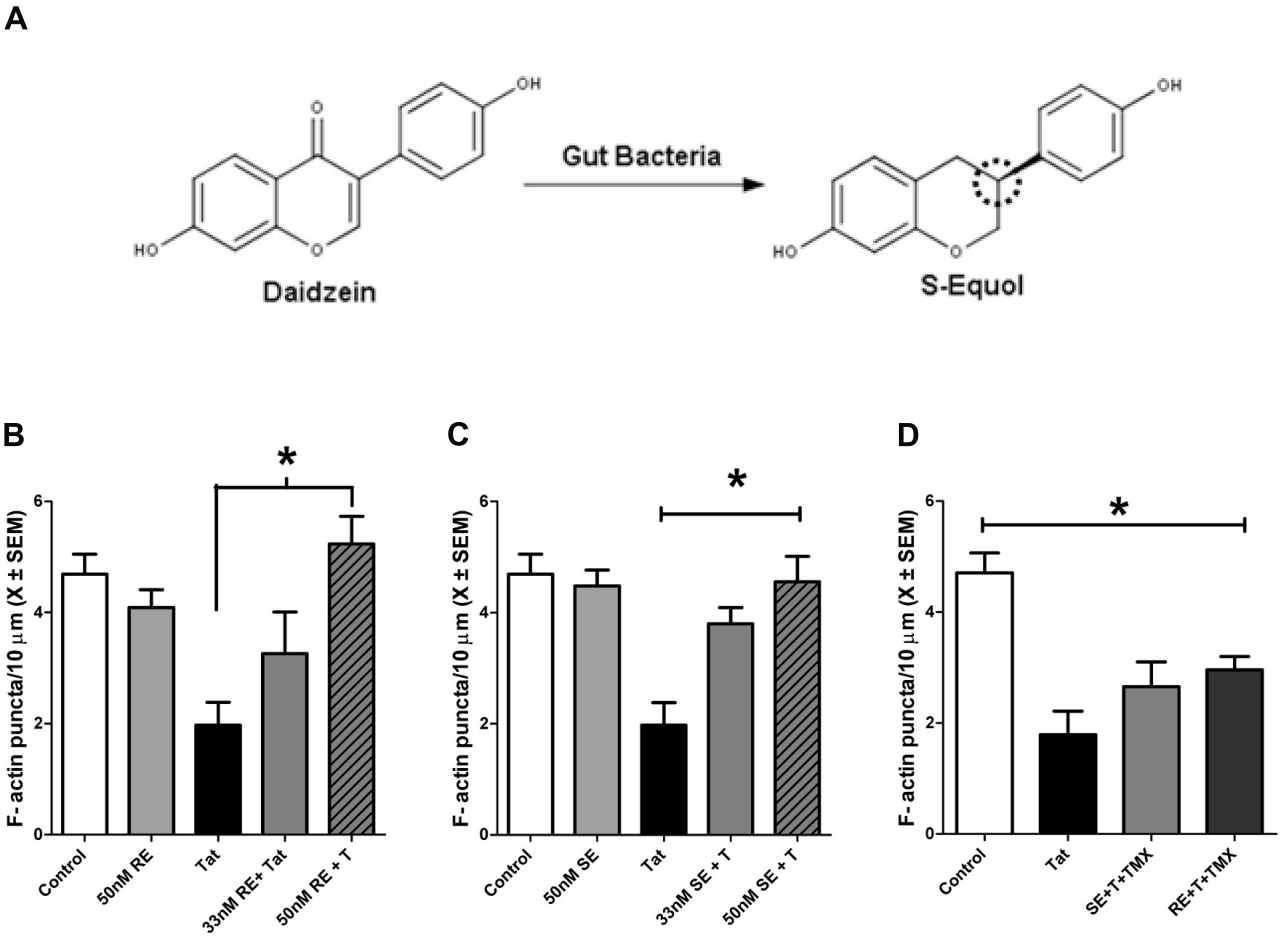
**R-Equol (RE) and S-Equol (SE) prevented synaptic loss induced by HIV-1 Tat (50 nM) via an estrogen receptor mediated mechanism. (A)** Conversion of the soy isoflavone daidzein to SE. The dotted circle identifies a chiral center on carbon 3 wherein a conformational change produces RE. However, only SE may be produced by mammalian gut bacteria. **(B)** A moderate concentration of RE (50 nM), but not a low concentration (33 nM), prevented significant F-actin puncta loss induced by HIV-1 Tat (50 nM; *p* ≤ 0.01). **(C)** Both the low (33 nM) and moderate (50 nM) concentrations of SE prevented HIV-1 Tat induced F-actin puncta loss (*p* ≤ 0.05 and *p* ≤ 0.001, respectively). **(D)** 1 h pre-treatment with TMX (100 nM) blocked the protective effects of RE and SE (50 nM) against HIV-1 Tat (50 nM) in midbrain neurons. Mean ± SEM, ^∗^*p* ≤ 0.05 compared to HIV-1 Tat treatment.

Midbrain neurons were pre-treated with either RE (33 or 50 nM) or SE (33 or 50 nM) for 24 h prior to 50 nM HIV-1 Tat (**Figure 1**). There was a significant main effect of treatment *F*(1,22) = 9.4, *p* ≤ 0.001, with 50 nM HIV-1 Tat producing a significant reduction in F-actin puncta (*p* ≤ 0.001). Pre-treatment with 50 nM RE (*p* ≤ 0.01), but not 33 nM RE, prevented significant loss of F-actin puncta following 50 nM HIV-1 Tat (**Figure 1B**). Pre-treatment with either 33 nM SE (*p* ≤ 0.05) or 50 nM SE (*p* ≤ 0.001) prevented HIV-1 Tat induced loss of F-actin puncta (**Figure 1C**). RE and SE treatment alone did not produce either significant loss or proliferation of F-actin puncta (**Figures 1B,C**). RE or SE pre-treated HIV-1 Tat (50 nM) neurons were not statistically different from vehicle-treated neurons.

### S-Equol and R-Equol Act Through an Estrogen Mediated Mechanism to Prevent HIV-1 Tat Induced Synapse Loss

The neuroprotective mechanisms of RE and SE in midbrain neurons against HIV-1 Tat induced synapse loss were determined by pre-treatment with TMX, a potent estrogen receptor antagonist. TMX was added to the cultures 1 h prior to the addition of either SE or RE. Cultures were then incubated with HIV-1 Tat 1–86 (50 nM) for 24 h. Pre-treatment with TMX prior to SE (*p* ≤ 0.02) and RE (*p* ≤ 0.02) resulted in significant loss of F-actin puncta relative to control cultures. These F-actin losses were not significantly different from cultures treated with HIV-1 Tat (50 nM) alone, suggesting that RE and SE act through an ER dependent mechanism (**Figure 1D**).

### HIV-1 Tat and Cocaine Interact to Produce Synaptopathy in Midbrain and Cortical Neurons

**FIGURE 2:**
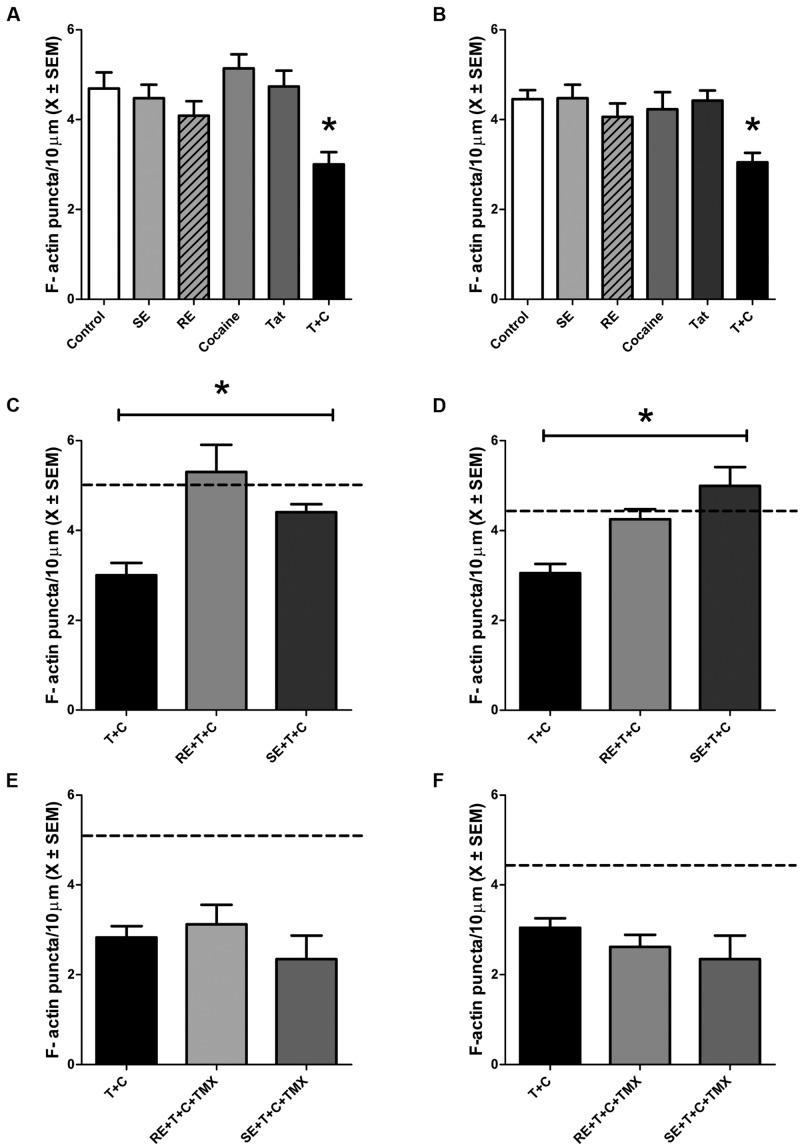
**HIV-1 Tat (10 nM) + cocaine (1.6 μM) treatment produced significant synaptic loss in midbrain and cortical neurons, which was prevented by pre-treatment with either RE or SE. (A)** Treatment with either SE, RE, HIV-1 Tat, or cocaine does not significantly alter the density of dendritic F-actin puncta compared to controls in midbrain neurons. Treatment with HIV-1 Tat + cocaine produced significant loss of F-actin puncta (*p* ≤ 0.05). **(B)** Treatment with either SE, RE, HIV-1 Tat, or cocaine does not significantly alter the density of dendritic F-actin puncta compared to controls in cortical neurons. Treatment with HIV-1 Tat + cocaine produced significant loss of F-actin puncta (*p* ≤ 0.05). **(C)** Pre-treatment with either RE or SE (50 nM) prevented dendritic F-actin puncta loss caused by HIV-1 Tat + cocaine treatments in midbrain neurons. Dendrites from pre-treated neurons are not significantly different from vehicle-treated controls (mean values, dotted line). **(D)** Pre-treatment with either RE or SE (50 nM) prevents HIV-1Tat + cocaine induced loss of dendritic F-actin puncta (*p* ≤ 0.001) in cortical neurons. Dendrites from pre-treated neurons are not significantly different from vehicle-treated controls (mean values, dotted line). **(E)** Pre-treatment of midbrain neurons with TMX (100 nM) for 1 h prior to either RE or SE prevented the protective effects of RE and SE, suggesting an estrogen receptor mediated mechanism. Vehicle-treated control mean values are represented by dotted line. **(F)** Pre-treatment of cortical neurons with TMX (100 nM) for 1 h prior to either RE or SE prevented the protective effects of RE and SE, suggesting an estrogen receptor mediated mechanism. Control mean value represented by dotted line. Mean ± SEM, ^∗^*p* ≤ 0.05 compared either to vehicle-treated controls **(A,B)** or HIV-1 Tat + cocaine **(C,D)**.

In order to examine the effects of HIV-1 Tat and cocaine on F-actin puncta, a low dose of HIV-1 Tat 1–86 (10 nM) and a physiologically relevant dose of cocaine (1.6 μM) were simultaneously added to the cultures and incubated for 24 h. As shown in **Figures 2A,B**, there was a significant effect of HIV-1 Tat + cocaine *F*(1,123) = 16.4, (*p* ≤ 0.001) but no significant effect of either HIV-1 Tat or cocaine individually on dendritic F-actin puncta. Neurons cultured from fetal cortical and midbrain regions responded to HIV-1 Tat + cocaine similarly, as there were no significant differences between the brain region cultured *F*(1,123) = 3.4, *p* > 0.05.

### S-Equol and R-Equol Prevent Interactive HIV-1 Tat + Cocaine Synaptopathy

Cortical and midbrain neurons were pre-treated with either 50 nM RE or SE for 24 h prior to HIV-1 Tat (10 nM) + cocaine (1.6 μM). There was a significant main effect of treatment *F*(5,123) = 7.6, *p* ≤ 0.001 (**Figures 2C,D**), but not a significant effect of brain region (*p* > 0.05). Using a Tukey *post hoc* analysis it was determined that HIV-1Tat + cocaine neurons pretreated with either RE or SE had significantly more dendritic F-actin puncta relative to HIV-1 Tat + cocaine treated neurons (*p* ≤ 0.001). The number of puncta on RE and SE pre-treated neurons and were not significantly different from vehicle-treated controls.

### S-Equol and R-Equol Prevent HIV-1 Tat + Cocaine Induced Synaptopathy via an ERβ Mechanism

Primary cortical and midbrain cultures were treated with TMX, an estrogen receptor antagonist, for 1 h prior to treatment with RE or SE. Following a 24 h incubation with HIV-1 Tat + cocaine, F-actin puncta were assessed. TMX pre-treatment prevented both RE and SE mediated synaptic protection following HIV-1 Tat + cocaine (*p* ≤ 0.01; **Figures 2E,F**).

**FIGURE 3:**
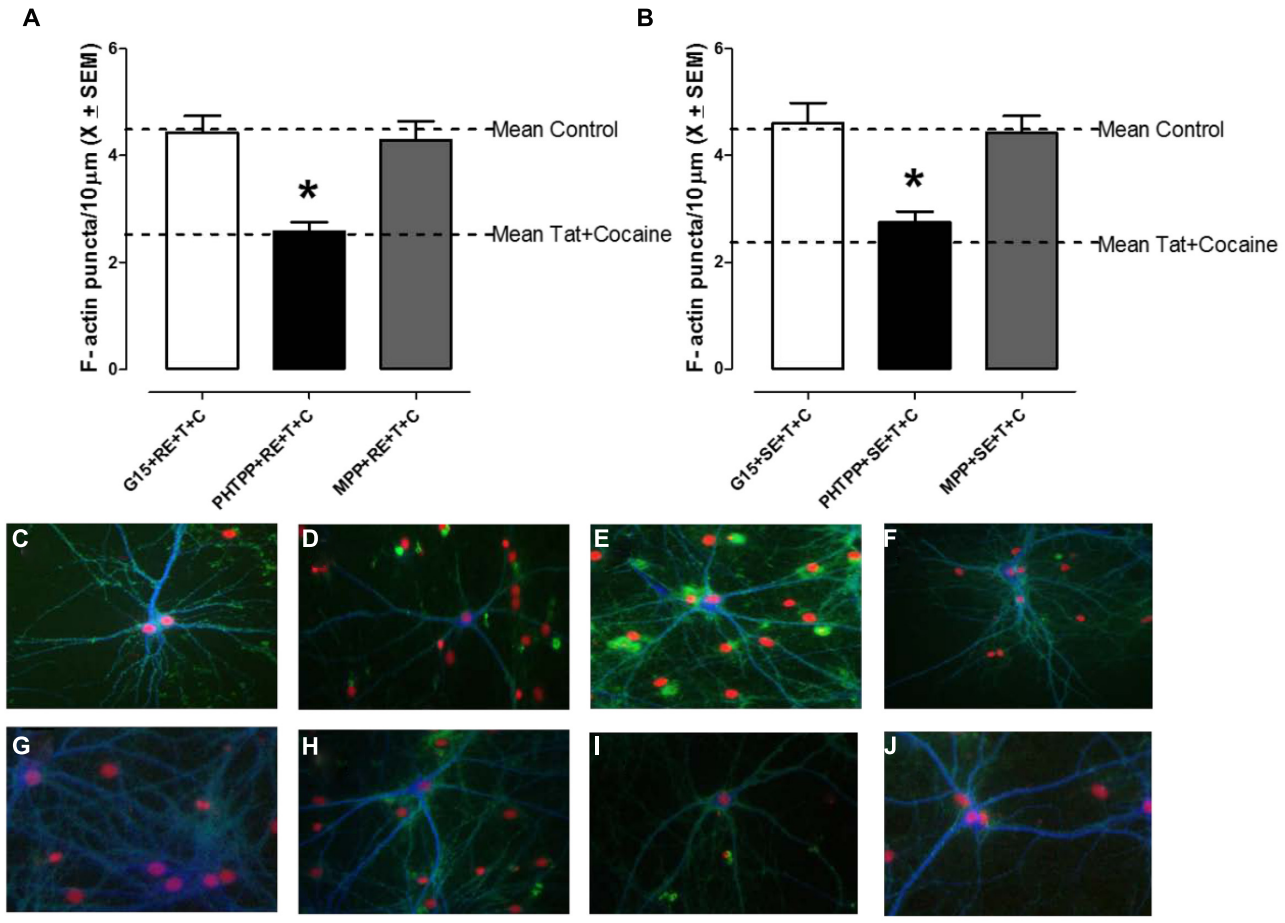
**R-Equol and SE prevented HIV-1 Tat + cocaine synaptic loss via a selective estrogen receptor (ERβ) dependent mechanism.** Cortical neurons were pre-treated for 1 h with either MPP (ERα), PHTPP (ERβ), or G15 (GPR30) prior to treatment with either RE **(A)** or SE **(B)** to identify which specific ER subtype was necessary for equol-mediated synaptic protection from HIV-1 Tat + cocaine. We found that pre-treatment with PHTPP (ERβ) inhibited both the RE (panel A) and SE (panel B) induced synaptic protection in HIV-1 Tat + cocaine treated cortical neurons. **(A)** HIV-1 Tat + cocaine exposed neurons pre-treated with either MPP or G15, and subsequently RE, had normal levels of dendritic F-actin puncta (i.e., not significantly different from vehicle-treated controls, *p* > 0.05). In contrast, PHTPP-treated neurons had a significant loss of dendritic F-actin puncta compared to controls (*p* ≤ 0.05) and were not significantly different from HIV-1 Tat + cocaine treated dendrites (*p* > 0.05), indicating that PHTPP inhibited RE-induced synaptic protection. Mean ± SEM, ^∗^*p* ≤ 0.05. **(B)** HIV-1 Tat + cocaine exposed neurons pre-treated with either MPP or G15, and subsequently SE, had normal levels of dendritic F-actin puncta (i.e., not significantly different from vehicle-treated controls, *p* > 0.05). In contrast, PHTPP-treated neurons had a significant loss of dendritic F-actin puncta compared to controls (*p* ≤ 0.05) and were not significantly different from HIV-1 Tat + cocaine treated dendrites (*p* > 0.05), indicating that PHTPP inhibited SE-induced synaptic protection. Mean ± SEM, ^∗^*p* ≤ 0.05. **(C–J)** The ERβ antagonist PHTPP significantly blocked equol-mediated protection from HIV-1 Tat + cocaine in cortical neurons. PHTPP pre-treated, SE**(I)**/RE**(J)** treated, neurons lacked a fine dendritic network and had reduced dendritic branching, similar characteristics to those in non pre-treated HIV-1Tat + cocaine neurons **(D)**. In contrast, vehicle **(C)**, HIV-1 Tat (10 nM; **G**), cocaine **(H)**, and SE**(E)**/RE**(F)** treated neurons had robust dendrites with complex branching patterns and a lack of aberrant morphology such as bundling or beading. Phalloidin/F-actin(green) and MAP-2 (blue); 20× magnification.

Cortical neurons were treated with one of three selective ER subtype antagonists, MPP (ERα), PHTPP (ERβ), or G15 (GPR30) for 1 h prior to treatment with RE or SE in order to identify the specific ER subtype required for equol-mediated synaptic protection following HIV-1 Tat + cocaine (**Figures 3A,B**). There was a significant main effect of antagonist treatment *F*(3,134) = 11.2, *p* ≤ 0.001. Further analysis revealed that both the MPP, as well as the G15, treated dendrites were not significantly different from vehicle-treated controls (*p* > 0.05); however, PHTPP-treated dendrites had a significant loss of F-actin puncta when compared to controls (*p* ≤ 0.05) and were not significantly different from HIV-1 Tat + cocaine treated dendtrites (*p* > 0.05). More specifically, only the ERβ antagonist PHTPP significantly prevented RE or SE mediated synaptic protection (*p* ≤ 0.001), indicative of an ERβ dependent mechanism. Examination of either vehicle (**Figure 3C**), HIV-1 Tat (10 nM; **Figure 3G**), cocaine (**Figure 3H**), or SE (**Figure 3E**)/RE (**Figure 3F**) pre-treated neurons found robust staining of dendrites with Phalloidin/F-actin (green) puncta and MAP-2 (blue) dendrites. These neurons exhibited complex dendritic branching patterns and an extensive fine dendritic network. In contrast, examination of PHTPP pre-treated SE/HIV-1 Tat + cocaine treated (**Figure 3I**), PHTPP pre-treated RE/HIV-1 Tat + cocaine treated (**Figure 3J**), and HIV-1 Tat + cocaine treated (**Figure 3D**) neurons found reduced dendritic branching, diminished Phalloidin staining, and dendritic fragmentation.

## Discussion

Neurocognitive deficits are more prevalent among HIV-1+ positive drug users (Nath, 2002; Levine et al., 2006; Devlin et al., 2012; Weber et al., 2013). Cocaine is one of the most commonly abused drugs in HIV-1+ individuals (Cook et al., 2007; Korthuis et al., 2008), and has been shown to potentiate neuronal death caused by HIV-1 proteins *in vitro* (Kendall et al., 2005; Aksenov et al., 2006). Although neuronal cell death is seen in HIV-1 (Li et al., 2005; Mattson et al., 2005), synaptic dysfunction and loss are more predictive of neurocognitive status (Ellis et al., 2007). We found (1) HIV-1 Tat + cocaine exposure resulted in a significant reduction in F-actin rich puncta/synapses, relative to either HIV-1 Tat or cocaine alone, (2) pre-treatment with either RE or SE prevented the HIV-1 Tat + cocaine induced puncta/synaptic loss, and (3) RE and SE provided synaptic protection from HIV-1 Tat + cocaine exposure via an ERβ dependent mechanism. These findings suggest that early treatment with RE or SE may prevent the synaptopathy caused by HIV-1 and cocaine abuse.

Cocaine potentiated the effects of HIV-1 Tat at doses in which HIV-1 Tat alone was not damaging to synaptic structures. The dose of HIV-1 Tat was fourfold lower than concentrations previously shown to cause neuronal death in similar neuronal cell cultures (Kendall et al., 2005). The dose of cocaine reflected physiological arterial levels observed in IV cocaine users (Evans et al., 1996) and rats (Mactutus et al., 1994; Booze et al., 1997); well below the cocaine concentration known to produce neuronal death *in vitro* (Bennett et al., 1993; Nassogne et al., 1995). However, low doses of HIV-1 Tat + cocaine produced synaptic loss at an early time point (24 h) prior to the time previously reported for neuronal death (48 h; Bertrand et al., 2014). HIV-1 Tat (Wallace et al., 2006; Ferris et al., 2009; Midde et al., 2013) and cocaine (Ritz and Kuhar, 1993) both inhibit dopamine transporter (DAT) function, increase oxidative stress (Aksenov et al., 2006; Fitting et al., 2015), and thereby may cause depolymerization of F-actin and a subsequent reduction of F-actin rich puncta/synapses.

S-Equol is the active metabolite of the soy derived phytoestrogen daidzein (Setchell and Cassidy, 1999; Jackson et al., 2011), and daidzein has been proposed to be responsible for the cognitive health benefits of soy products (Setchell and Cassidy, 1999; Lampe, 2009; Jackson et al., 2011). Daidzein is metabolized into SE via two distinct steps by various bacteria found in the gut of animals and humans (Setchell et al., 2005; Jackson et al., 2011). In humans, daidzein supplementation results in plasma concentrations of 8–20 ng/ml S-equol (see review: Jackson et al., 2011). A low concentration of S- and RE (33 nM) as well as the mid-range concentration of equol (50 nM) are equivalent to plasma concentrations observed in humans following supplementation (8 and 12 ng/ml, respectively); hence, these doses were used to evaluate potential synaptic protection. Both enantiomers achieve full synaptic protection at physiologically relevant concentrations that were 20x lower than that of daidzein required for full protection (Bertrand et al., 2014). Neurons pre-treated with daidzein and exposed to a neurotoxic concentration of HIV-1 Tat protein were able to not only maintain, but also restore lost synapses (Bertrand et al., 2014); together, these results and those in the present study, suggest that SE may be a potent aid in the prevention and restoration of synaptic loss.

Both equol enantiomers prevented HIV-1 Tat-induced (50 nM) and HIV-1 Tat (10 nM) + cocaine induced synaptic loss. TMX pre-treatment blocked the neuroprotective actions of both RE and SE, indicating that equol mediated neuroprotection was via an estrogen receptor dependent mechanism. SE is a potent ER agonist, with preferential binding to ERβ over ERα (Setchell et al., 2005; Lampe, 2009; Jackson et al., 2011). In order to determine the mechanism of both S- and RE synaptic protection, the compounds MPP, PHTPP, and G15 (ERα, ERβ, and GPR30 antagonists, respectively) were used to determine which ER subtype mediated the neuroprotective effects of SE and RE. Only PHTPP blocked the neuroprotective effects of SE and RE, indicating that both enantiomers provide synaptic protection via an ERβ dependent mechanism.

Our prior studies have found that treatment with 17β-estradiol mitigates interactions between HIV-1 Tat protein and cocaine, thereby preventing neuronal cell death in an ER dependent manner (Kendall et al., 2005). Treatment with 17β-estradiol suppresses HIV-1 Tat enhancement of LTR promoter activity in astrocytes (Wilson et al., 2006). Over expression of ERα prevents 17β-estradiol mediated suppression of Tat-LTR promoter activity in astrocytes (Heron et al., 2009). Unfortunately, activation of the ERα subtype may lead to unwanted side effects, limiting the use of specific agonists which act at ERα receptors. We reported estrogenic activation of the ERβ prevented HIV-1 Tat-induced apoptosis (Adams et al., 2010, 2012), and the present findings suggest ERβ is necessary for synaptic protection following HIV-1 Tat and cocaine. Thus, an ERβ specific compound (i.e., SE or other compounds), may be beneficial for both preventing HIV-1 synaptopathy, although the functional consequences of ERβ specific compounds in HIV-1 therapeutics remains to be determined.

The gut-brain axis is a new area of study which recognizes the bidirectional communication between the gut microbiota and behavior (Cryan and Dinan, 2012; Foster and McVey Neufeld, 2013). SE is produced by the gut microbiota in humans and animals, but not all humans are SE producers. Up to 80% of individuals in China, Japan, and Korea are capable of producing SE (Morton et al., 2002; Fujimoto et al., 2008), however, only 25% of the population that consumes a Western diet are SE producers (Lampe et al., 1998). It is currently unknown how HIV-1 may affect the gut microbiota, however, it may play an important role in mediating disease progression as well as the neurocognitive correlates of HIV-1. The ability of the gut metabolite SE to alter the synaptopathy induced either by HIV-1 Tat alone, or HIV-1 Tat and cocaine in combination, suggests that the gut microbiota could play an important role in modulating CNS health in HIV-1.

HIV-1 protein Tat in combination with cocaine resulted in significant loss of synapses; loss which may play a role in the increased prevalence of HAND in cocaine users (Nath, 2010; Purohit et al., 2011). Cognitive function in HAND is closely correlated with synaptodendritic injury (Ellis et al., 2007), and a reduction in synapses represents a potential mechanism for the increased prevalence of HAND in HIV-1+ cocaine users (Martin-Thormeyer and Paul, 2009; Nath, 2010; Gill and Kolson, 2014). HIV-1 Tat + cocaine synaptopathy occurs prior to overt neuronal death (Kim et al., 2008; Bertrand et al., 2013, 2014), and the synaptopathy is potentially reversible (Bertrand et al., 2014), highlighting the importance of compounds, like SE, that may offer synaptic protection and restoration of normal synaptic connectivity. Interestingly, we found that the synaptic protective actions of equol may be mediated by an ERβ-dependent mechanism. Equol actions targeted to the ERβ may limit unwanted side-effects, yet still provide neuroprotection at a level similar to the effects of 17β-estradiol (Kendall et al., 2005; Simpkins and Singh, 2008; Adams et al., 2010; Simpkins et al., 2012). SE is more potent than the parent compound daidzein (Adams et al., 2012; Bertrand et al., 2014), suggesting that either direct treatment with SE or treatment via gut metabolism of daidzein may promote synaptic complexity following HIV-1 Tat or Tat + cocaine exposure. Overall, the present data suggest that ERβ may be a novel therapeutic target for the treatment of HAND + drug abuse synaptopathy and early therapeutic intervention with SE may prevent HIV-1 synaptopathy and potentially delay the development of HAND.

## Conflict of Interest Statement

The authors declare that the research was conducted in the absence of any commercial or financial relationships that could be construed as a potential conflict of interest.
